# The impact of Karnofsky performance status on prognosis of patients with hepatocellular carcinoma in liver transplantation

**DOI:** 10.1186/s12876-024-03161-7

**Published:** 2024-02-26

**Authors:** Jie Zhou, Danni Ye, Siyao Zhang, Jiawei Ding, Tao Zhang, Zheng Chen, Fangshen Xu, Shenli Ren, Zhenhua Hu

**Affiliations:** 1 Division of Hepatobiliary and Pancreatic Surgery, Department of Surgery, First Afliated Hospital, School of Medicine, Zhejiang University, Hangzhou, China; 2 Division of Hepatobiliary and Pancreatic Surgery, Department of Surgery, Fourth Affiliated Hospital, School of Medicine, Zhejiang University, Yiwu, China

**Keywords:** Liver transplantation, Hepatocellular carcinoma, Karnofsky Performance Status scale, Waiting list, Intent-to-treat survival, Tumor recurrence

## Abstract

**Background:**

Functional performance as measured by the Karnofsky Performance Status (KPS) scale has been linked to the outcomes of liver transplant patients; however, the effect of KPS on the outcomes of the hepatocellular carcinoma (HCC) liver transplant population has not been fully elucidated. We aimed to investigate the association between pre-transplant KPS score and long-term outcomes in HCC patients listed for liver transplantation.

**Methods:**

Adult HCC candidates listed on the Scientific Registry of Transplant Recipients (SRTR) database from January 1, 2011 to December 31, 2017 were grouped into group I (KPS 80–100%, *n* = 8,379), group II (KPS 50–70%, *n* = 8,091), and group III (KPS 10–40%, *n* = 1,256) based on percentage KPS score at listing. Survival was compared and multivariable analysis was performed to identify independent predictors.

**Results:**

Patients with low KPS score had a higher risk of removal from the waiting list. The 5-year intent-to-treat survival was 57.7% in group I, 53.2% in group II and 46.7% in group III (*P* < 0.001). The corresponding overall survival was 77.6%, 73.7% and 66.3% in three groups, respectively (*P* < 0.001). Multivariable analysis demonstrated that KPS was an independent predictor of intent-to-treat survival (*P* < 0.001, reference group I; HR 1.19 [95%CI 1.07–1.31] for group II, *P* = 0.001; HR 1.63 [95%CI 1.34–1.99] for group III, *P* < 0.001) and overall survival(*P* < 0.001, reference group I; HR 1.16 [95%CI 1.05–1.28] for group II, *P* = 0.004; HR 1.53 [95%CI 1.26–1.87] for group III, *P* < 0.001). The cumulative 5-year recurrence rates was higher in group III patients (7.4%), compared with 5.2% in group I and 5.5% in group II (*P* = 0.037). However, this was not significant in the competing regression analysis.

**Conclusions:**

Low pre-transplant KPS score is associated with inferior long-term survival in liver transplant HCC patients, but is not significantly associated with post-transplant tumor recurrence.

**Supplementary Information:**

The online version contains supplementary material available at 10.1186/s12876-024-03161-7.

## Background

Hepatocellular carcinoma (HCC) is a major malignancy that ranks fourth for cancer-related mortality in the world [1]. Because of the presence of underlying liver disease such hepatitis B virus (HBV) and hepatitis C virus (HCV) infections, alcoholic liver disease, and nonalcoholic steatohepatitis (NASH), patients with HCC often complicate with ascites, malnutrition, and sarcopenia, induced by cirrhosis, and present with diminished liver function and poor functional status.

Liver transplantation (LT) provides the only curative treatment for HCC patients with impaired liver function for whom hepatectomy is not feasible. Functional status is one of the important predictors of mortality in HCC patients listed for LT [2]. Functional status is determined by a variety of factors including patient age and nutritional status. Karnofsky Performance Status (KPS) scale was one of the various methods which have been incorporated into the investigation of functional status in cirrhotic patients. Indeed, previous studies have shown that poor KPS score was associated with increased waiting list mortality in liver transplant candidates, which is independent of liver disease severity determined by laboratory MELD score [2, 3]. Patient functional status is also an independent predictor of post-transplant mortality [4].

The use of the KPS in predicting outcomes in candidate HCC patients listed for LT has not been fully examined. One study based on the United Network for Organ Sharing (UNOS) database evaluated the association of KPS and wait-list mortality in patients with and without HCC [3]. A more recent study based on a database from the United Kingdom and Ireland assessed the time-dependent impact of functional status on LT outcomes for patients with and without HCC [5]. However, they censored patient survival at the time of transplantation to follow-up for 1 year, which was a relatively short period and which might not have reflected the long-term impact of functional status. Another study found that KPS was an independent predictor for bone metastases of HCC patients underlying LT, but the study was limited by its small sample size, which was from a single center [6]. The use of the KPS in predicting outcomes in HCC candidates listed for LT has not been fully examined.

Our current study aimed to investigate the association between pre-transplant KPS and long-term post-transplant outcomes in candidates with HCC, using data from the SRTR database. We attempted to evaluate the impact of KPS on long-term outcomes including intent-to-treat survival and overall survival, as well as on post-transplant tumor recurrence, to provide useful evidence for clinical practice.

## Methods

This study used data from the SRTR, the data system that includes data on all donors, wait-listed candidates, and transplant recipients in the United States (US) submitted by the members of the Organ Procurement and Transplantation Network (OPTN). The Health Resources and Services Administration and the US Department of Health and Human Services provide oversight to the activities of the OPTN and SRTR contractors. The data reported here have been supplied by the Hennepin Healthcare Research Institute as the contractor for the SRTR. The interpretation and reporting of these data are the responsibility of the author(s) and in no way should be seen as an official policy of or interpretation by the SRTR or the US government. The protocol for the present study was reviewed and approved by the Ethics Committee of Zhejiang University, China.

**Fig. 1:**
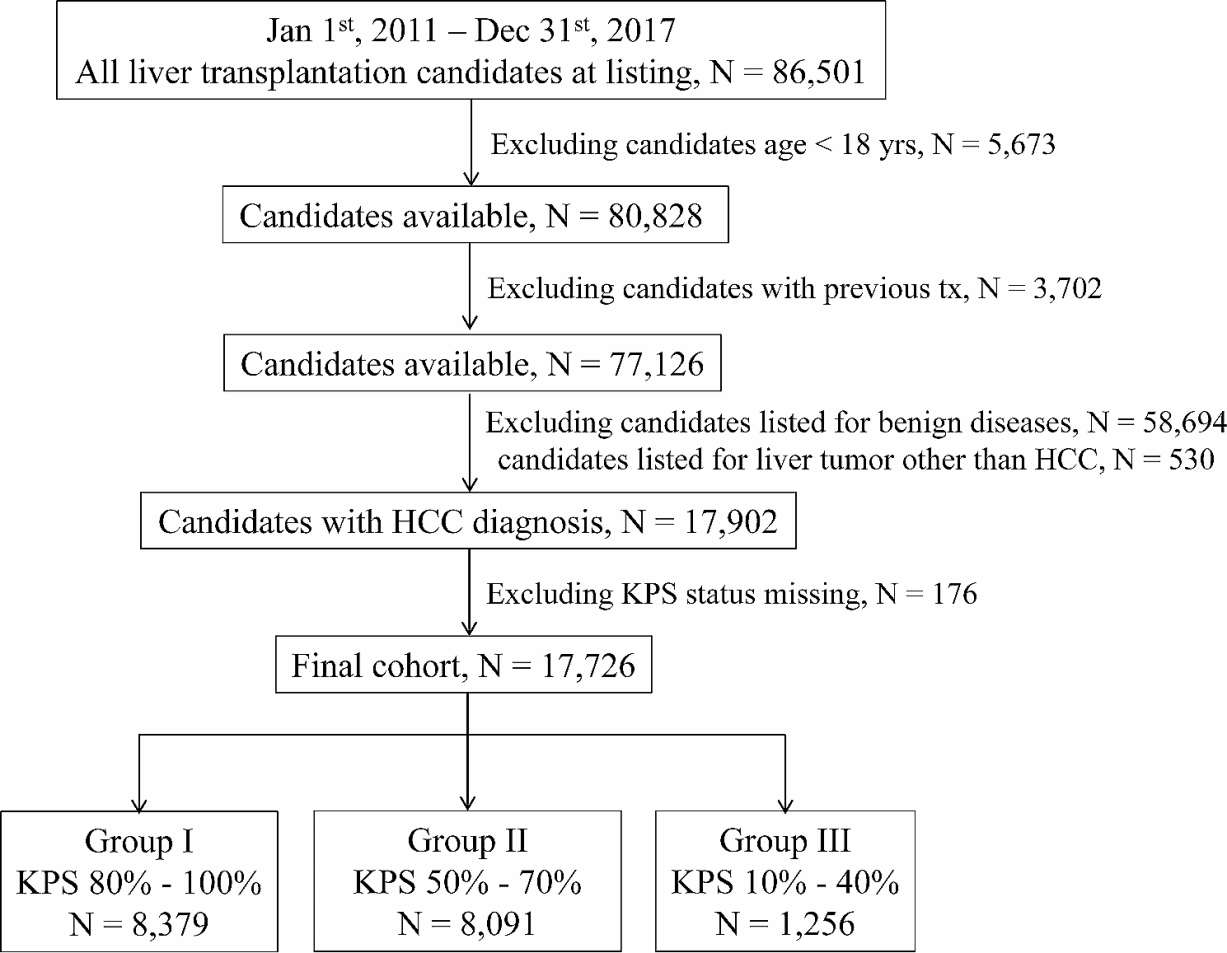
Flow chart. *HCC*, hepatocellular carcinoma; *KPS*, Karnofsky performance status

We retrospectively included candidates with HCC wait-listed for LT between January 1, 2011 and December 31, 2017. The inclusion criteria were candidate age ≥ 18 years and a primary diagnosis of “hepatocellular carcinoma” or “hepatoma” at listing. The exclusion criteria were as follows: (1) candidate age < 18 years old; (2) candidates with a previous LT; (3) candidates with a primary diagnosis of benign liver diseases; (4) candidates with a liver tumor other than HCC; and (5) candidates with missing pre-transplant KPS status. We finally included 17,726 patients in our current study, and divided them into three groups according to KPS status, which was based on the candidate’s ability to work or care for themselves as follows: group I, KPS range from 80 to 100%, *N* = 8,379; group II, KPS range from 50 to 70%, *N* = 8,091; and group III, KPS range from 10 to 40%, *N* = 1,256. The candidate selection process is shown in Fig. 1 in detail.

We first compared baseline characteristics that included both candidate variables and donor variables (for transplanted recipients) among the three groups.

We then investigated the cumulative dropout rate from the waiting list among the three groups, which was calculated from the date of listing to the date of the patients’ removal from the waiting list because of death, disease deterioration, or medical unsuitability.

The primary endpoint of this study was long-term outcomes of HCC patients, which included intent-to-treat survival (analyzed from the date of listing) and overall survival (analyzed from the date of transplantation), and was compared among three groups. We furthered performed univariate and multivariable analysis to identify independent predictors for intent-to-treat survival and overall survival. The secondary endpoint of the study was post-transplant HCC recurrence, which was defined according to previous studies by Samoylova et al. [7] and Orci et al. [8]; And cumulative HCC recurrence rates were also compared among the three groups.

### Statistical analysis

Continuous variables were analyzed using Kruskal-Wallis test and were reported as means and standard deviations, or medians and inter-quartile ranges (IQRs) where appropriate. Categorical variables were analyzed using chi-square test and reported as counts and proportions. The intent-to-treat survival and overall survival were analyzed using the Kaplan-Meier method and compared using the log-rank test. We used the Cox proportional regression hazard ratios (HRs) model with the forward likelihood method to identify the association between KPS status and intent-to-treat survival, as well as overall survival. The time-dependent effects of KPS for survival were investigated based on Schoenfeld’s residuals [9, 10]. The cumulative dropout rate and the HCC recurrence rate were investigated with a competing risk model. Competing risk regression analysis was performed to evaluate the association between KPS and tumor recurrence, with death as the competing risk [11]. The statistical significance was set at a two-tailed *P* value < 0.05. Analyses were performed using SPSS version 22.0 (IBM, Armonk, NY, United States) and R for Windows (version 4.0.2).

## Results

### Baseline characteristics

The mean KPS score was 71.97% (standard deviation, 16.94%). The proportion of candidates in the three KPS categories remained similar year by year throughout the study period (Supplementary Fig. 1). Significant differences were observed between the three groups for the candidate variables of sex, age, race, underlying liver disease, life support on ventilator, laboratory MELD score, serum albumin, serum bilirubin, serum INR, serum creatinine, and serum sodium, whereas ABO blood type and BMI were similar among groups. In terms of tumor variables, candidates in group III tended to have larger tumor size and also received less pre-transplant treatments including TACE, RFA, and surgery. However, tumor number, pre-transplant AFP level, proportion of group within Milan criteria, and treatment including chemotherapy and cryoablation were similar among the three groups. In terms of donor variables in transplanted patients, group III tended to be younger in donor age and have more male donors, whereas donor race, ABO blood type, cause of death, and DCD status were similar among groups. The detailed information for the three groups is shown in Table 1.

**Table 1:** Baseline characteristics

	KPS I(N =8,379)	KPS II(N =8,091)	KPS III(N =1,256)	P value
**Candidate characteristics**				
Sex				<0.001
M	6634 (79.2%)	6042 (74.7%)	931 (74.1%)	
F	1745 (20.8%)	2049 (25.3%)	325 (25.9%)	
Age	60.13 ± 7	60.03 ± 6.82	58.99 ± 7.1	<0.001
Race				<0.001
White	5324 (63.5%)	5094 (63%)	843 (67.1%)	
Black or African American	891 (10.6%)	809 (10%)	134 (10.7%)	
Asian	814 (9.7%)	529 (6.5%)	44 (3.5%)	
Hispanic/Latino	1240 (14.8%)	1557 (19.2%)	215 (17.1%)	
Other	110 (1.3%)	102 (1.3%)	20 (1.6%)	
ABO				0.456
A	3096 (36.9%)	2968 (36.7%)	490 (39%)	
B	1122 (13.4%)	1024 (12.7%)	157 (12.5%)	
O	3829 (45.7%)	3788 (46.8%)	565 (45%)	
AB	332 (4%)	311 (3.8%)	44 (3.5%)	
Underlying liver disease				<0.001
HCV	5013 (59.8%)	4837 (59.8%)	696 (55.4%)	
HBV	709 (8.5%)	469 (5.8%)	60 (4.8%)	
Alcohol	813 (9.7%)	1056 (13.1%)	202 (16.1%)	
NASH	725 (8.7%)	734 (9.1%)	122 (9.7%)	
Other	1119 (13.4%)	995 (12.3%)	176 (14%)	
Ventilator	1 (0%)	2 (0%)	51 (4.1%)	<0.001
BMI	28.79 ± 5.2	28.96 ± 5.42	28.83 ± 5.81	0.231
MELD	10.26 ± 3.73	11.94 ± 5.1	19.58 ± 9.95	<0.001
Albumin	3.4 ± 0.7	3.23 ± 0.71	3.11 ± 0.72	<0.001
Bilirubin	2.55 ± 4.99	3.47 ± 6.28	8.66 ± 11.63	<0.001
INR	1.38 ± 0.66	1.53 ± 0.89	2.02 ± 1.38	<0.001
Creatinine	1.04 ± 0.87	1.18 ± 0.99	1.68 ± 1.45	<0.001
Sodium	137.87 ± 4.03	137.3 ± 4.51	136.32 ± 5.57	<0.001
**Tumor characteristics**				
Treatment				
TACE	5214 (66.8%)	4873 (66.9%)	461 (58.1%)	<0.001
RFA	768 (9.8%)	527 (7.2%)	54 (6.8%)	<0.001
Chemotherapy	17 (0.2%)	14 (0.2%)	5 (0.6%)	0.047
Cryoablation	275 (3.5%)	237 (3.3%)	24 (3%)	0.565
Surgery	133 (1.7%)	74 (1%)	10 (1.3%)	0.001
Tumor Staging				0.092
within Milan	7223 (97%)	6689 (96.7%)	716 (95.6%)	
beyond Milan	223 (3%)	229 (3.3%)	33 (4.4%)	
Tumor nubmer	1.2 ± 0.51	1.21 ± 0.53	1.24 ± 0.55	0.132
Largest tumor diameter (cm)	1.39 ± 1.57	1.44 ± 1.45	1.63 ± 1.47	<0.001
Sum of tumor diameters (cm)	1.69 ± 1.97	1.76 ± 1.89	2.01 ± 1.95	<0.001
AFP value (ng/ml)	110 ± 812.81	130.29 ± 885.06	95.93 ± 527.13	0.402

*AFP*, Alpha-fetoprotein; *BMI*, body mass index; *CNS*, central nervous system; *DCD*, donation after cardiac death; *HBV*, hepatitis B virus; *HCV*, hepatitis C virus; *INR*, international normalized ratio; *KPS*, Karnofsky Performance Status; *MELD*, Model for End-stage Liver Diseases; *NASH*, nonalcoholic steatohepatitis; *RFA*, radiofrequency ablation; *TACE*, transarterial chemoembolization

### Cumulative dropout rate from waiting list

The median time from date of listing to dropout from waiting list was 6 months (IQR 3–12 months). The cumulative 1-year, 3-year, and 5-year dropout rates for group III candidates was 27.3%, 30.2%, and 31.0% respectively, significantly higher than those of group I, at 19.1%, 27.3%, and 28.6%, respectively and group II, at 22.8%, 29.5%, and 30.1%, respectively (Supplement Fig. 2; *P* < 0.001).

**Fig. 2:**
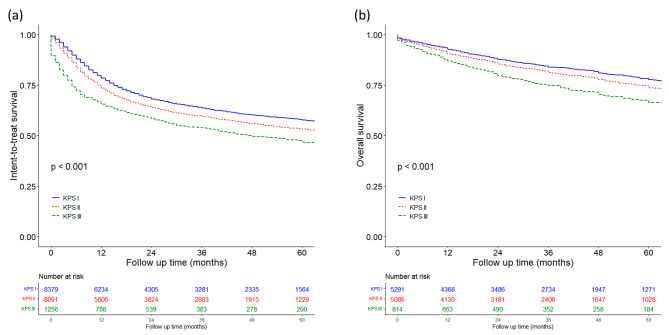
Long-term outcomes of HCC patients in each Karnofsky performance status group: **(a)** intent-to-treat survival; **(b)** overall survival

### Intention-to-treat survival

The 1-, 3-, and 5-year intent-to-treat survival for group I was 78.4%, 63.7%, and 57.7%, respectively, which was significantly better than that of group II, with corresponding survival of 73.5%, 59.5%, and 53.2%, and group III, with corresponding survival of 65.5%, 53.7%, and 46.7%, respectively (Fig. 2a; *P* < 0.01).

In the univariable analysis, candidate age, KPS, race, ABO blood type, underlying liver disease, life support on ventilator, BMI, laboratory MELD score, tumor number, largest tumor diameter, sum of tumor diameters, pre-transplant AFP level, tumor within Milan criteria, donor age, donor race, donor ABO blood type, and donor cause of death were observed to be associated with intent-to-treat survival and were further evaluated in the multivariable analysis (Supplementary Table 1).

In the multivariable analysis, KPS status (*P* < 0.001, reference KPS I; HR 1.19 [95%CI 1.07–1.31] for KPS II, *P* = 0.001; HR 1.63 [95%CI 1.34–1.99] for KPS III, *P* < 0.001), as well as candidate age, candidate race, candidate BMI, sum of tumor diameter, pre-transplant AFP, and donor age, was an independent predictor for intent-to-treat survival. (Table 2).

**Table 2:** Multivariable analysis for intent-to-treat survival

	HR (95%CI)	***P*** value
Candidate age	1.02 (1.01–1.03)	< 0.001
KPS (ref. I)		< 0.001
KPS II	1.19 (1.07–1.31)	0.001
KPS III	1.63 (1.34–1.99)	< 0.001
Candidate race (ref. White)		0.002
Black or African American	1.13 (0.97–1.31)	0.122
Asian	0.67 (0.54–0.83)	< 0.001
Hispanic/Latino	0.94 (0.82–1.08)	0.374
Other	0.90 (0.56–1.45)	0.656
Candidate BMI	0.99 (0.98–0.10)	0.003
Sum of tumor diameter	1.07 (1.05–1.09)	< 0.001
Pre-transplant AFP	2.23 (1.78–2.79)	< 0.001
Donor age	1.01 (1.00–1.01)	< 0.001

*AFP*: Alpha-fetoprotein; *BMI*: body mass index; *HR*: hazard ratio; *KPS*: Karnofsky Performance Status

We further evaluated the time-dependent effect of KPS on intent-to-treat survival, which showed a generally stable HR, according to Cox-derived estimates, during the follow-up years (Supplementary Fig. 3a).

### Overall survival

The 1-, 3-, and 5-year overall survival for group I was 92.6%, 83.9%, and 77.6%, respectively, which was significantly better than that of group II which had a corresponding survival of 90.4%, 81.2%, and 73.7%, respectively, followed by group III with corresponding survival of 86.9%, 74.7%, and 66.3%, respectively (Fig. 2b, *P* < 0.001).

In the univariate analysis, we observed that candidate age, KPS status, race, ABO blood type, laboratory MELD score, tumor number, largest tumor diameter, sum of tumor diameters, pre-transplant AFP level, tumor within Milan criteria, donor age, donor ABO blood type, and donor cause of death were associated with overall survival, which we further took into the multivariable analysis (Supplementary Table 2).

In the multivariable analysis, KPS status (*P* < 0.001, reference KPS I; HR 1.16 [95%CI 1.05–1.28] for KPS II, *P* = 0.004; HR 1.53 [95%CI 1.26–1.87] for KPS III, *P* < 0.001), as well as candidate age, candidate race, sum of tumor diameter, pre-transplant AFP, donor ABO blood type, and donor age, was an independent predictor for overall survival (Table 3).

**Table 3:** Multivariable analysis for overall survival

	HR (95%CI)	***P*** value
Recipient age	1.02 (1.01–1.03)	< 0.001
KPS (ref. I)		< 0.001
KPS II	1.16 (1.05–1.28)	0.004
KPS III	1.53 (1.26–1.87)	< 0.001
Candidate race (ref. White)		0.032
Black or African American	1.16 (0.10–1.35)	0.058
Asian	0.77 (0.62–0.96)	0.021
Hispanic/Latino	0.97 (0.85–1.12)	0.714
Other	0.89 (0.55–1.44)	0.645
Sum of tumor diameter	1.06 (1.04–1.08)	< 0.001
Pre-transplant AFP	2.20 (1.76–2.76)	< 0.001
Donor ABO (ref. A)		0.022
B	0.86 (0.73–1.01)	0.058
O	1.08 (0.97–1.20)	0.146
AB	0.91 (0.69–1.21)	0.527
Donor age	1.01 (1.00–1.01)	< 0.001

*AFP*: Alpha-fetoprotein; *BMI*: body mass index; *HR*: hazard ratio; *KPS*: Karnofsky Performance Status

We also evaluated the time-dependent effect of KPS on overall survival, which also showed a stable HR during the follow-up years (Supplementary Fig. 3b).

### Post-transplant hepatocellular carcinoma recurrence

**Fig. 3:**
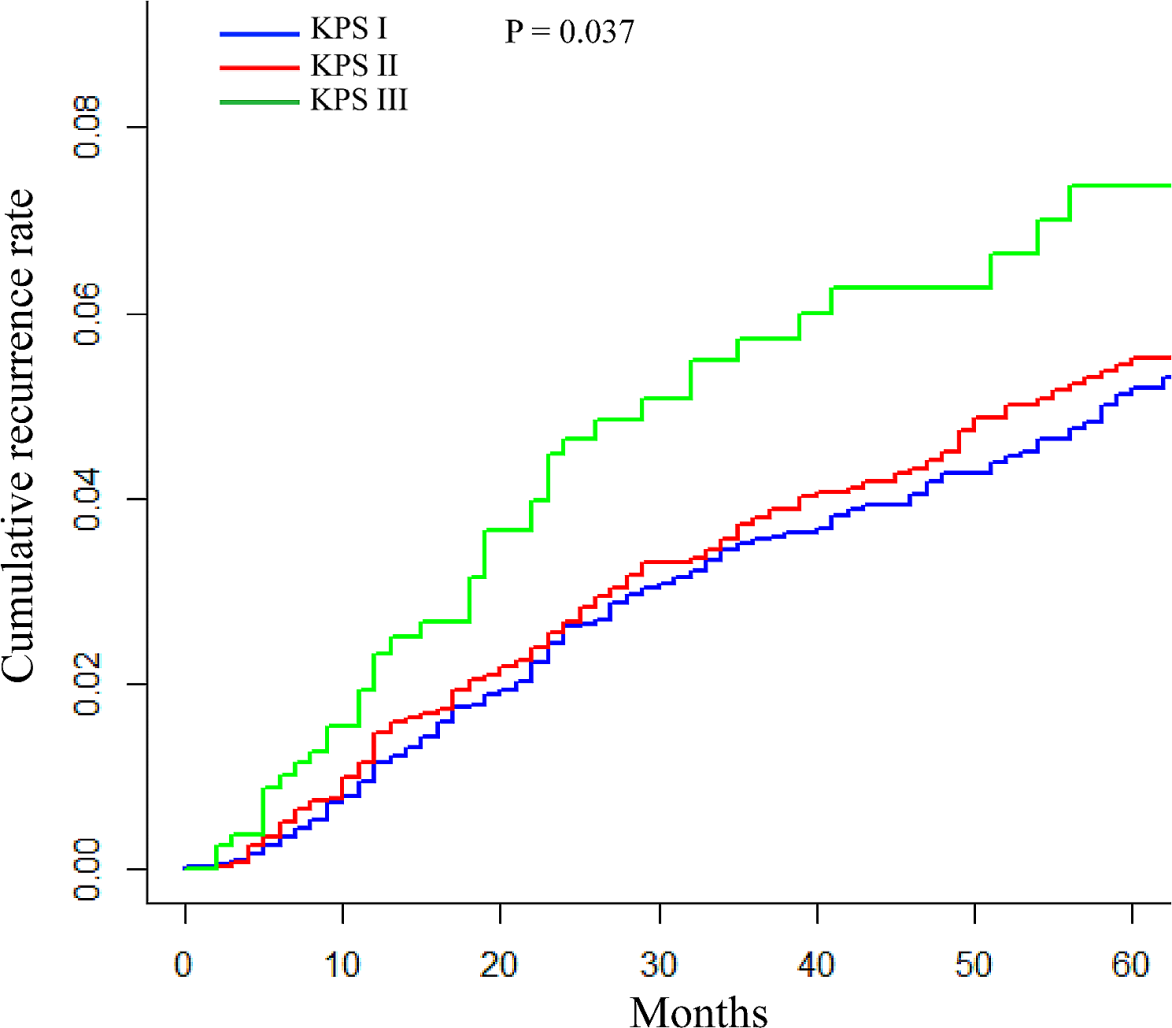
Post-transplant cumulative tumor recurrence rate. *KPS*, Karnofsky performance status

The median time from LT to recurrence was 20 months (IQR 11–33 months). The cumulative 1-year, 3-year, and 5-year recurrence rates in patients with KPS III was 2.3%, 5.7%, and 7.4%, respectively, which were significantly higher than those of KPS I and KPS II, with corresponding recurrence rates of 1.2%, 3.6%, and 5.2% and 1.5%, 3.8%, and 5.5% respectively (Fig. 3, *P* = 0.037). In the competing regression risk model, worsening KPS was associated with higher probability of tumor recurrence in the univariate analysis (*P* = 0.037, reference KPS I; HR 1.07 [95%CI 0.87–1.31] for KPS II, *P* = 0.53; HR 1.55 [95%CI 1.11–2.16] for KPS III, *P* = 0.01). However, significant associations were not seen in the multivariable analysis (data not shown).

## Discussion

In this study, we have shown that a lower pre-transplant KPS score was associated with higher waiting list mortality and was an independent predictor for both inferior intent-to-treat survival and OS in patients with HCC awaiting or having had LT, after adjustment for other clinical risk factors. A lower KPS score was also associated with a higher probability of post-transplant HCC recurrence, but did not reach significance in the multivariable competing risk regression analysis.

These findings add to the current knowledge about the impact of functional status on LT in the HCC patient. The role of KPS in LT has been emphasized in recent years. The previous liver transplant allocation system was based on the severity of candidate liver disease as determined by laboratory MELD score. Although the MELD score was objective, it did not include clinical variables that may affect transplant outcomes. With the aging of the population, there is also an increasing proportion of candidates of older age listed for LT [12, 13]. Older candidates have less physiologic reserve and are more likely to have clinical complications, including sarcopenia and malnutrition, which makes them more vulnerable to stress. This may lead to disadvantage in survival for patients waiting for LT. Thus there is an urgent need to co-opt the factors reflecting patient frailty status to better predict survival in LT patients.

Previous studies have already shown that poor pre-transplant KPS was associated with increased mortality in patients with cirrhosis on the LT waiting list, especially in those without HCC [9]. In another study, poor, as well as unknown, pre-transplant KPS was also associated with post-transplant mortality [14]. The study by Thulavath et al. [15] also evaluated the dynamic change in KPS before and after LT, and found it an independent predictor for graft and patient survival. Their group also investigated the dynamic change in KPS following LT in patients with acute on chronic liver failure (ACLF) and found that good performance improvements in these patients may also be a consideration in clinical practice of whether to allocate a donor liver to a patient with multi-organ failure [16].

Attempts have been made to develop and use other prognostic measurements of frailty in patients with cirrhosis, including objective tools such as the ECOG functional status, 6-minute walk test, sarcopenia (determined by psoas muscle area), and the Fried model of frailty [2, 17–20]. However, the information gained on their application in epidemiological studies of potential LT candidates has been limited.

The KPS has also been utilized in oncological practice to predict prognosis. Excellent inter-rater reliability and reproducibility have been achieved in clinical practice [21–23]. The KPS has been included in the scoring system developed for patients with HCC by one French group, as one of the five independent predictors for prognosis [24]. A later study also validated the prognostic value of this system from a Chinese HCC cohort, which showed superior predictive value over other scoring systems such as CLIP score, CUPI, JIS score, and AJCC TNM classification [25]. The author concluded that the inferiority in the discrimination of later systems was possibly because they do not include functional status, which is important to establishing prognosis in HCC patients.

In the setting of LT, the study by Orman et al. found that higher KPS score was observed in HCC patients and was also significantly associated with waiting list mortality. Yet the relationship of KPS with transplantation rate was only significant in univariable analysis. These observations are also consistent with current guidelines that favor the HCC population with better functional status as reflected by lower native laboratory MELD scores. The use of other functional status evaluation systems has also been assessed in LT for HCC patients. A recent study by Wallace et al. assessed the time-dependent impact of functional status, as stratified by ECOG scale, on outcomes after LT, and found that it was not associated with 1-year post-transplant survival for HCC patients.

Our study demonstrated that poor functional status, as determined by KPS score, was significantly associated with higher likelihood of removal from the waiting list, which is consistent with the observation by Orman et al. [3]. However, we observed that lower KPS score was not only associated with long-term intent-to-treat survival but also with long-term overall survival. Also, pre-transplant KPS was an independent prognostic factor for both intent-to-treat survival and overall survival. This is different to Wallace et al. [5], which may be due to the differing demographics of UK and US HCC patients, as well as the different functional status assessment tools used in the studies. In the study by Wallace et al. [5], functional status was determined by ECOG scale score, which was stratified into five levels. The HCC patients in the lowest level showed a trend of inferior survival probability; however, because the number of patients in this group was small compared with other the groups, evaluating performance-specific difference was difficult, as the authors suggested [21]. Our study included 17,726 HCC patients listed for LT in the US, which was larger than the UK cohort, and would make statistical evaluation of the impact of functional status more robust.

We also observed that lower KPS score was associated with increased probability of post-transplant tumor recurrence, especially in KPS III patients. However, this was only significant in the univariable competing regression analysis, not in the multivariable analysis. Tumor recurrence was one of the most important factors impairing post-transplant outcomes in HCC patients. Previous recipient selection criteria have been established based on tumor characteristics including tumor number, tumor size, and tumor biology reflected by AFP level, which are closed related to tumor recurrence [26–28]. In our study, although KPS was not independently associated with tumor recurrence, lower KPS impaired long-term survival of HCC patients, after adjusting for other confounders including tumor characteristics. This suggests the potential importance of the management of functional status in HCC patients. As HCC patients tend to be in a better functional state with more preserved liver function at listing, we have previously placed more emphasis on tumor characteristics to select appropriate patients. However, with the aging of the general population, more aged HCC patients than ever are being listed for LT, and are more likely to have accompanying complications brought on by frailty. This impaired functional status might persist during the post-transplant period and would impair survival in the follow-up period.

The current findings underscore the importance of assessment of functional status in HCC candidates in addition to conventional clinical risk factors, to better predict post-transplant survival for those patients. Patients of low KPS are more likely to drop out of the waiting list and are less likely to be considered for LT. Factoring in the aging population, we might expect a trend of more removals from the waiting list and fewer transplants in those patients [3]. Meticulous pre-transplant evaluation in addition to intervention such as exercise therapy and nutritional support to improve patient functional status is needed. The study by Lai et al. analyzed data from the Functional Assessment in Liver Transplantation (FrAILT) Study and found that pre-transplant frailty status worsened in the 3 months after LT, making patients vulnerable to early post-transplant death and increasing the length of hospital stay [29]. Although this status did improve modestly within 12 months, less than 40% of patients achieved a robust status, which ultimately adversely affected post-transplant outcomes. With regard to this, pre-transplant assessment of functional status has been suggested for incorporation into the decision-making procedure for LT, to indicate the suitability of candidates at listing, as well as guide prehabilitative intervention for individual candidates to achieve better transplant outcomes [30].

Our study has several limitations. First, the assessment of KPS is based on patient report or clinician evaluation, which may vary between observers and raises concern of lower reliability than objective assessments such as 6-minute walk test and muscle bulk assessment using radiography. However, previous studies have verified the excellent inter-rater reliability of the KPS scale in many clinical settings. Objective assessments may be less cost-effective or require more specific training and need to be validated in clinical practice. Indeed, the KPS has been collected by the SRTR for more than 10 years and its advantages in terms of simplicity and validity have been demonstrated. Second, as the KPS is only a clinical observational assessment of functional status, we could not investigate the underlying mechanisms that influenced KPS status, despite the importance of this in informing further modifications to pre-transplant nutritional and physical therapies for candidate status improvement. Despite these limitations, our study provides a comprehensive investigation of the influence of pre-transplant KPS on HCC patients listed for LT in the US, based on a large sample from the SRTR database, which adds new insights into decision making on LT for HCC patients.

## Conclusions

Low pre-transplant KPS score is associated with inferior long-term survival for HCC patients undergoing LT; however, it is not significantly associated with post-transplant tumor recurrence. These findings indicate the importance of measurement of functional status as the initial tool in evaluating the suitability of candidates for LT. The feasibility of adopting such tools into risk-prediction models needs further assessment. Further studies aimed at assessing feasible multidisciplinary interventions to improve functional status in a prospective manner are needed, to achieve better long-term prognosis for HCC patients.

### Electronic supplementary material

Below is the link to the electronic supplementary material.

Supplementary Material 1

Supplementary Material 2

## Data Availability

The datasets used and/or analysed during the current study are available from the corresponding author on reasonable request.

## Abbreviations

ACLF: Acute-on-chronic liver failure
AFP: Alpha-fetoprotein
BMI: Body mass index
CNS: Central nervous system
DCD: Donation after cardiac death
HBV: Hepatitis B virus
HCC: Hepatocellular carcinoma
HCV: Hepatitis C virus
HRs: Hazard ratios
KPS: Karnofsky Performance Status
HHRI: Hennepin Healthcare Research Institute
HRSA: Health Resources and Services Administration
IQRs: Inter-quartile ranges
ITS: Intent-to-treat survival
LT: Liver transplantation
MELD: Model for end-stage liver disease
NASH: Nonalcoholic steatohepatitis
OPTN: Organ Procurement and Transplantation Network
OS: Overall survival
RFA: Radiofrequency ablation
SRTR: Scientific Registry of Transplant Recipients
TACE: Transarterial chemoembolization
UNOS: United Network for Organ Sharing
US: United States
