# Supplementary material for: The impact of Karnofsky performance status on prognosis of patients with hepatocellular carcinoma in liver transplantation
Source: BMC Gastroenterol. 2024 Feb 26;24:85. doi: 10.1186/s12876-024-03161-7 (PMC10895807; doi:10.1186/s12876-024-03161-7)
Supplement: Supplementary file 1 — Supplementary Material 1 [file 12876_2024_3161_MOESM1_ESM.docx]

**Supplement Table 1. Univariate analysis for intent-to-treat survival**

|  |  | HR (95%CI) | P value |
| --- | --- | --- | --- |
| Candidate characteristics | Age | 1.01 (1.01 - 1.02) | <0.001 |
|  | Gender (female vs. male) | 0.10 (0.97 - 1.03) | 0.835 |
|  | KPS (ref. KPS I) |  | <0.001 |
|  | KPS II | 1.19 (1.13 - 1.25) | <0.001 |
|  | KPS III | 1.53 (1.40 - 1.68) | <0.001 |
|  | Race (ref. White) |  | <0.001 |
|  | Black or African American | 1.12 (1.03 - 1.20) | 0.005 |
|  | Asian | 0.86 (0.79 - 0.95) | 0.002 |
|  | Hispanic/Latino | 1.10 (1.03 - 1.17) | 0.004 |
|  | Other | 1.07 (0.87 - 1.32) | 0.527 |
|  | ABO (ref. A) |  | <0.001 |
|  | B | 0.87 (0.80 - 0.94) | <0.001 |
|  | O | 1.07 (1.02 - 1.13) | 0.009 |
|  | AB | 0.76 (0.66 - 0.88) | <0.001 |
|  | Underlying liver disease (ref. HCV) |  | <0.001 |
|  | HBV | 0.77 (0.69 - 0.86) | <0.001 |
|  | Alcohol | 1.74 (1.62 - 1.86) | <0.001 |
|  | NASH | 1.16 (1.07 - 1.27) | 0.001 |
|  | Other | 1.67 (1.56 - 1.78) | <0.001 |
|  | Ventilator | 2.73 (1.96 - 3.81) | <0.001 |
|  | BMI | 0.99 (0.99 - 0.10) | 0.008 |
|  | MELD | 1.03 (1.03 - 1.04) | <0.001 |
| Tumor characteristics | Tumor Staging (beyond vs. within Milan) |  |  |
|  | beyond Milan | 1.67 (1.47 - 1.89) | <0.001 |
|  | Tumor number | 1.19 (1.14 - 1.25) | <0.001 |
|  | Largest tumor diameter (cm) | 1.09 (1.07 - 1.10) | <0.001 |
|  | Sum of tumor diameters (cm) | 1.08 (1.07 - 1.09) | <0.001 |
|  | AFP value (≥400 vs. <400 ng/ml) | 2.66 (2.43 - 2.93) | <0.001 |
| Donor characteristics | Donor age | 1.01 (1.00 - 1.01) | <0.001 |
|  | Race (ref. White) |  | <0.001 |
|  | Black or African American | 1.07 (0.95 - 1.20) | 0.257 |
|  | Asian | 0.95 (0.72 - 1.24) | 0.684 |
|  | Hispanic/Latino | 1.04 (0.91 - 1.19) | 0.569 |
|  | Other | 12.27 (11.49 - 13.09) | <0.001 |
|  | ABO (ref. A) |  | 0.026 |
|  | B | 0.86 (0.747 – 1.00) | 0.05 |
|  | O | 1.07 (0.98 - 1.18) | 0.151 |
|  | AB | 1.03 (0.80 - 1.32) | 0.837 |
|  | Gender (female vs. male) | 1.00 (0.92 - 1.10) | 0.949 |
|  | Cause of death (ref. anoxia) |  | 0.036 |
|  | Cerebrovascular/stroke | 1.16 (1.04 - 1.29) | 0.008 |
|  | Head trauma | 1.01 (0.90 - 1.13) | 0.855 |
|  | CNS tumor | 0.84 (0.42 - 1.68) | 0.612 |
|  | Other | 1.24 (0.91 - 1.70) | 0.18 |
|  | DCD | 1.14 (0.96 - 1.35) | 0.127 |
|  | BMI | 1.01 (1.00 - 1.01) | 0.063 |
| *AFP*, Alpha-fetoprotein; *BMI,* body mass index*; CNS*, central nervous system ;*DCD,* donor after cardiac death; *HBV,* hepatitis B virus; *HCV,* hepatitis C virus; *HR,* hazard ratio; *INR,* international normalized ratio; *KPS*, Karnofsky Performance Status; *MELD,* Model for End-stage Liver Diseases; *NASH,* nonalcoholic steatohepatitis; *RFA,* radiofrequency ablation; *TACE,* transarterial chemoembolization. | | | |

**Supplement Table 2. Univariate analysis for overall survival**

|  |  | HR (95%CI) | P value |
| --- | --- | --- | --- |
| Recipient characteristics | Age | 1.01 (1.01 - 1.02) | <0.001 |
|  | Gender (female vs. male) | 0.95 (0.90 - 1.01) | 0.077 |
|  | KPS (ref. KPS I) |  | <0.001 |
|  | KPS II | 1.21 (1.10 - 1.33) | <0.001 |
|  | KPS III | 1.67 (1.43 - 1.94) | <0.001 |
|  | Race (Ref. White) |  | <0.001 |
|  | Black or African American | 1.17 (1.02- 1.34) | 0.026 |
|  | Asian | 0.69 (0.57 - 0.85) | <0.001 |
|  | Hispanic/Latino | 0.93 (0.82 - 1.05) | 0.246 |
|  | Other | 0.72 (0.45- 1.16) | 0.176 |
|  | ABO (ref. A) |  | 0.013 |
|  | B | 0.87 (0.75 - 1.00) | 0.057 |
|  | O | 1.07 (0.97 - 1.18) | 0.176 |
|  | AB | 0.86 (0.69 - 1.08) | 0.2 |
|  | Underlying liver disease (ref. HCV) |  | 0.158 |
|  | HBV | 0.83 (0.69 - 0.99) | 0.039 |
|  | Alcohol | 1.02 (0.86 - 1.20) | 0.86 |
|  | NASH | 1.08 (0.92 - 1.27) | 0.362 |
|  | Other | 1.07 (0.93 - 1.24) | 0.357 |
|  | Ventilator | 1.74 (0.91 - 3.35) | 0.097 |
|  | BMI | 0.99 (0.98 – 1.00) | 0.055 |
|  | MELD | 1.02 (1.02 - 1.03) | <0.001 |
| Tumor characteristics | Tumor Staging (beyond vs. within Milan) |  | <0.001 |
|  | beyond Milan | 1.66 (1.31 - 2.10) |  |
|  | Tumor number | 1.16 (1.07 - 1.25) | <0.001 |
|  | Largest tumor diameter (cm) | 1.07 (1.05 - 1.09) | <0.001 |
|  | Sum of tumor diameters (cm) | 1.07 (1.05 - 1.09) | <0.001 |
|  | AFP value (≥400 vs. <400 ng/ml) | 2.15 (1.73 - 2.68) | <0.001 |
| Donor characteristics | Donor age | 1.01 (1.00 - 1.01) | <0.001 |
|  | Race (ref. White) |  | 0.659 |
|  | Black or African American | 1.05 (0.93 - 1.18) | 0.418 |
|  | Asian | 0.98 (0.75 - 1.28) | 0.885 |
|  | Hispanic/Latino | 1.09 (0.96 - 1.25) | 0.182 |
|  | Other | 1.14 (0.74 - 1.75) | 0.561 |
|  | ABO (ref. A) |  | 0.005 |
|  | B | 0.83 (0.72 - 0.96) | 0.014 |
|  | O | 1.08 (0.98 - 1.19) | 0.136 |
|  | AB | 0.94 (0.74 - 1.21) | 0.648 |
|  | Gender (female vs. male) | 1.00 (0.91 - 1.09) | 0.921 |
|  | Cause of death (ref. anoxia) |  | 0.04 |
|  | Cerebrovascular/stroke | 1.15 (1.04 - 1.28) | 0.01 |
|  | Head trauma | 1.00 (0.90 - 1.13) | 0.941 |
|  | CNS tumor | 0.84 (0.42 - 1.70) | 0.634 |
|  | Other | 1.23 (0.89 - 1.68) | 0.208 |
|  | DCD | 1.11 (0.94 - 1.32) | 0.226 |
|  | BMI | 1.01 (1.00 - 1.01) | 0.092 |
| *AFP*, Alpha-fetoprotein; *BMI,* body mass index*; CAN*, central nervous system; *DCD,* donor after cardiac death; *HBV,* hepatitis B virus; *HCV,* hepatitis C virus; *HR,* hazard ratio; *INR,* international normalized ratio; *KPS*, Karnofsky Performance Status; *MELD,* Model for End-stage Liver Diseases; *NASH,* nonalcoholic steatohepatitis; *RFA,* radiofrequency ablation; *TACE,* transarterial chemoembolization. | | | |
