# Supplementary material for: The impact of Karnofsky performance status on prognosis of patients with hepatocellular carcinoma in liver transplantation
Source: BMC Gastroenterol. 2024 Feb 26;24:85. doi: 10.1186/s12876-024-03161-7 (PMC10895807; doi:10.1186/s12876-024-03161-7)
Supplement: Supplementary file 2 — Supplementary Material 2 [file 12876_2024_3161_MOESM2_ESM.docx]

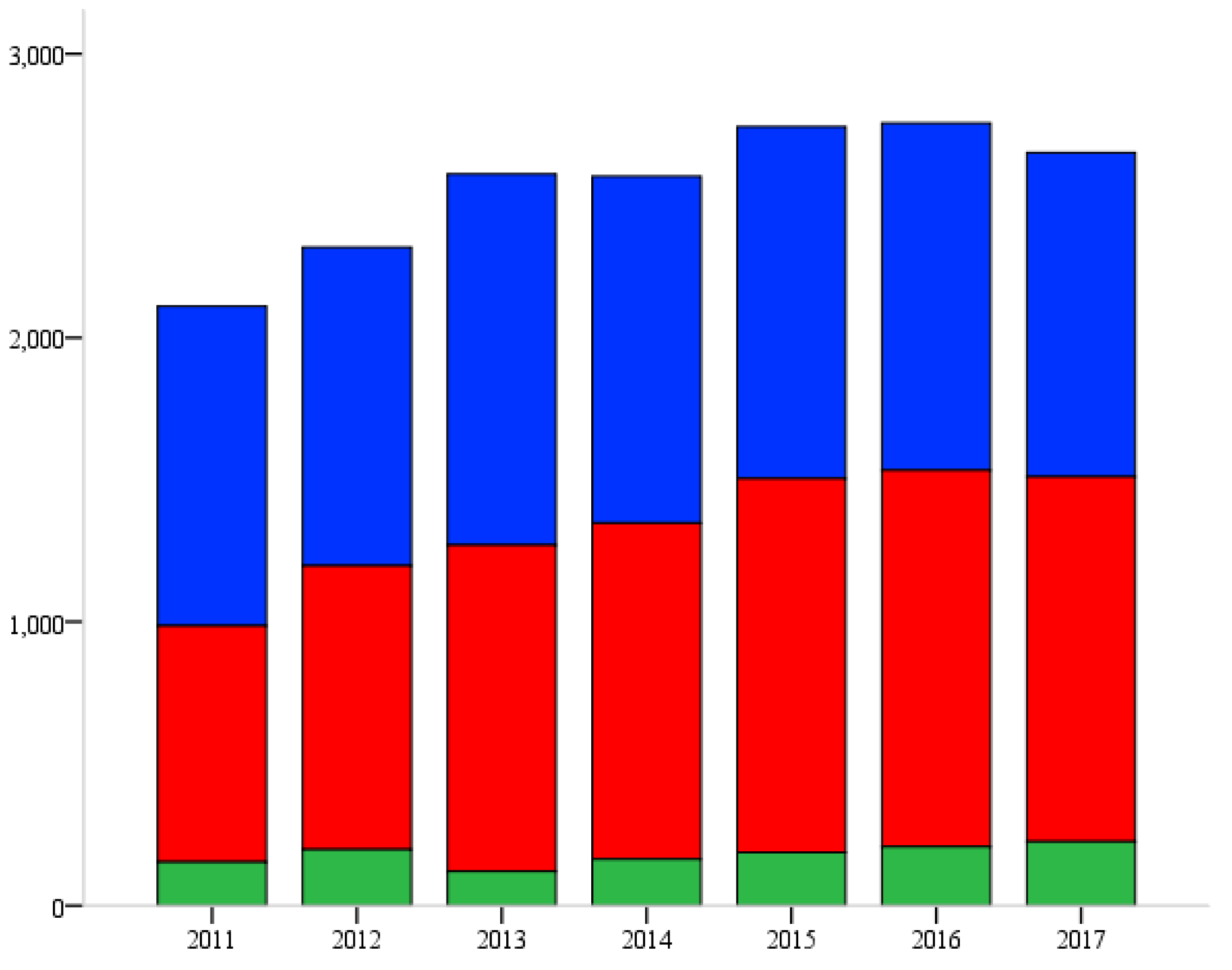


Supplementary Figure 1. Proportion of candidates in each Karnofsky Performance Status group listed by transplant year.


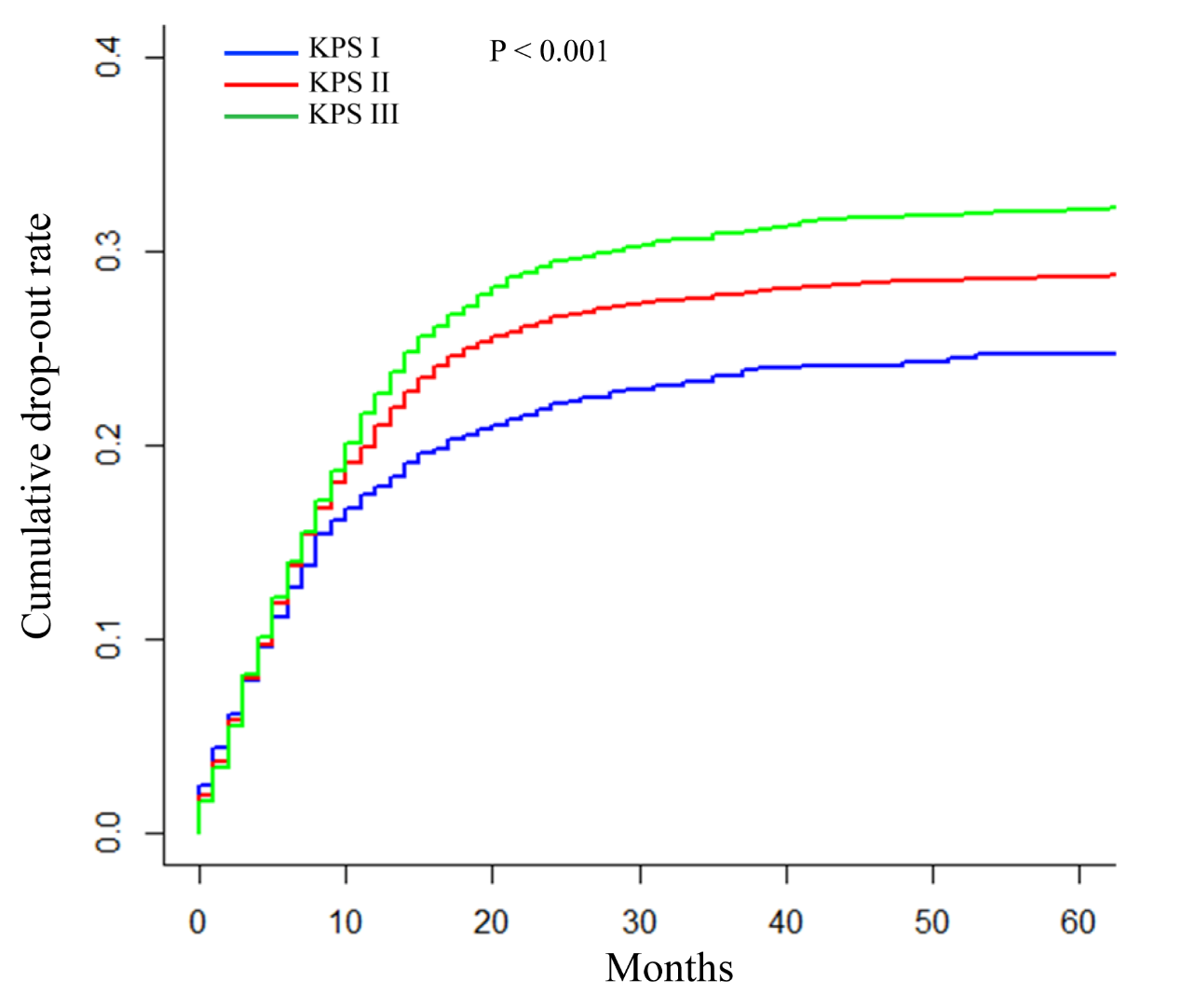


Supplementary Figure 2. Cumulative dropout rate. *KPS,* Karnofsky Performance Status.


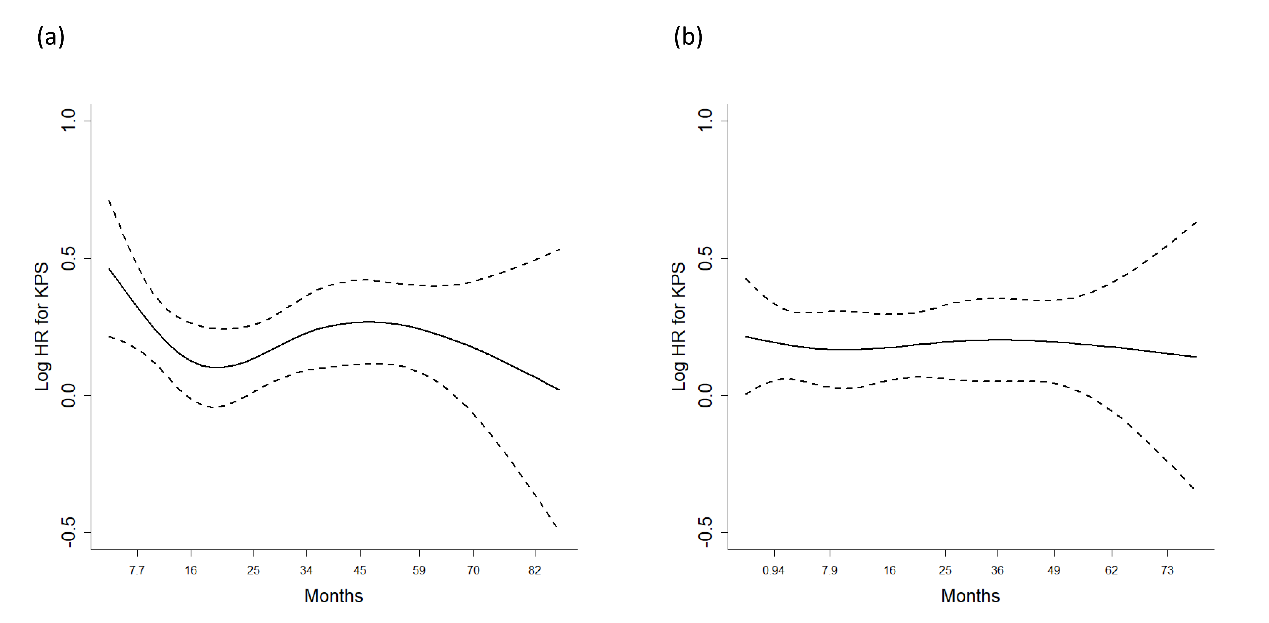


Supplementary Figure 3. Time-dependent effects for survival: (a). intent-to-treat survival; (b). overall survival. *KPS,* Karnofsky Performance Status.
